# Five-year trends in overweight and obesity among preschool children before and during the COVID-19 period: a retrospective repeated-measures analysis in Southwest China

**DOI:** 10.3389/fpubh.2026.1842094

**Published:** 2026-07-17

**Authors:** Peiling Cai, Tao Deng, Zhen Yang, Zhanyi Xu, Chengwei Zhang, Fanli Zhou, Yurong Li, Yuxuan Liu, Xiaoming Feng, Rui Huang

**Affiliations:** 1 School of Preclinical Medicine and School of Nursing, Chengdu University, Chengdu, Sichuan, China; 2 Department of General Practice, The General Hospital of PLA Western Theater Command, Chengdu, China; 3 Maternal and Child Health Service Center of Wuhua District, Kunming, Yunnan, China; 4 Clinical Medical College & Affiliated Hospital of Chengdu University, Chengdu, Sichuan, China; 5 Department of Pediatrics, Zhongshan Hospital of Xiamen University, School of Medicine, Xiamen University, Xiamen, China

**Keywords:** COVID-19, generalized linear mixed model, obesity, overweight, preschool children, repeated-measures analysis, school closure

## Abstract

**Background:**

COVID-19-related school closures and subsequent control measures may have altered children’s daily routines and contributed to changes in weight status. However, evidence on sex-specific and age-specific patterns of overweight and obesity among preschool children across both pre-pandemic and COVID-19 periods remains limited. This study aimed to examine five-year trends in overweight and obesity among preschool children in Southwest China from 2018 to 2022.

**Methods:**

This retrospective repeated-measures analysis used annual routine health examination records of preschool children aged 3.0 to 7.0 (exclusive) years in Wuhua District, Kunming, Yunnan Province, Southwest China, from 2018 to 2022. Annual examinations were conducted during the same calendar window each year, from May 25 to June 30. In 2020, this window corresponded to the initial kindergarten reopening period in Kunming after COVID-19-related school closures. The full analytic dataset was used to describe annual patterns in overweight, obesity, and combined overweight and obesity (OwO). Children with at least two annual health examination records were further included in generalized linear mixed model (GLMM) analyses. Overweight, obesity, and OwO were modeled as binary outcomes, respectively. Year of examination was included as the main fixed effect, with 2020 as the reference year, and child ID was included as a random effect. Age and sex were adjusted in the overall models, and age was adjusted in sex-stratified models.

**Results:**

A total of 17,561 children contributing 26,246 annual health examination records were included in the full analytic dataset. The repeated-measures subset included 7,175 children contributing 15,860 records. In the full analytic dataset, obesity prevalence was highest in 2020, whereas overweight and OwO prevalence were highest in 2022. Among boys, obesity prevalence peaked in 2020 at 4.7%. Among girls, overweight, obesity, and OwO were highest in 2022, reaching 12.1, 2.9, and 15.1%, respectively. In the overall GLMM analyses, the odds of overweight were higher in 2022 than in 2020 (*OR*: 1.92, 95% CI: 1.26–2.92), while the odds of obesity were lower in 2018, 2019, and 2021 than in 2020. The odds of OwO were lower in 2018, 2019, and 2021 than in 2020, but higher in 2022 (*OR*: 1.82, 95% CI: 1.14–2.89). Sex-stratified GLMM analyses showed that the higher obesity burden around 2020 was more evident among boys, whereas girls showed higher odds of overweight, obesity, and OwO in 2022.

**Conclusion:**

Preschool children in Southwest China showed heterogeneous temporal patterns of overweight and obesity before and during the COVID-19 period. Obesity was more prominent around the initial kindergarten reopening period in 2020, particularly among boys, whereas overweight, obesity and OwO were higher in 2022, particularly among girls. These findings support continued monitoring of preschool children’s weight status and the implementation of targeted early-life obesity prevention strategies following major public health disruptions.

## Introduction

Childhood overweight and obesity have emerged as a critical challenge for global public health, closely associated with a wide range of both short-term and long-term psychosocial impairments and non-communicable diseases (NCDs) (1). These include diminished self-esteem, peer relationship difficulties, poor academic achievement and peer problems (2), depression (3), as well as cardiovascular diseases (4), type 2 diabetes (T2D) (5), dyslipidemia (6), fatty liver disease (7), cancer (8), multiple sclerosis (MS) (9), Crohn’s disease (10), early-onset polycystic ovary syndrome (PCOS) in girls (11, 12), asthma (13) and skeletal deformities (1).

The preschool period represents a crucial window for growth monitoring and preventive interventions (14, 15), as patterns established during this early-life stage often track into later childhood and even adulthood, influencing lifelong health trajectories. Overweight and obesity in childhood have a high probability of persisting into adulthood (16, 17), significantly increasing the risk of T2D (18, 19) and elevating cardiovascular disease mortality (18, 19) in adulthood. Ultimately, this leads to a substantially heightened lifelong burden of NCDs (20). During this stage, dietary patterns, physical activity habits, sleep rhythms, and sedentary behaviors are still developing and are strongly shaped by family and kindergarten environments. The kindergarten setting may help support healthy weight-related behaviors by providing structured daily routines, opportunities for physical activity, and nutritionally regulated meals (21, 22). Disruptions to these routines may therefore have important implications for energy balance and weight status among preschool children.

The unprecedented coronavirus disease 2019 (COVID-19) pandemic and associated public health measures substantially reshaped children’s daily routines, including decreased engagement in daily physical activity, notably moderate-to-vigorous physical activity (MVPA) (23–25), increased sedentary time (24, 25), prolonged screen time (25), shifted sleep schedules with delayed bedtime and wake-up time leading to circadian rhythms disturbances (26, 27), as well as imbalances in energy intake and expenditure, marked by heightened consumption of energy-dense, ultra-processed foods and diminished intake of fruits and vegetables (28). Such lifestyle changes may have contributed to a marked increase in the prevalence of overweight and obesity among preschool children during the pandemic (29–33), and may have further exacerbated the global burden of childhood obesity.

However, the magnitude and persistence of these changes may vary by sex, age, local prevention policies, and the timing of school closure and reopening. Several evidence gaps remain. First, existing studies have primarily focused on relatively short observation windows during lockdown or shortly after school reopening, whereas fewer studies have examined trends across both pre-pandemic years and subsequent pandemic-control years. Second, crude annual comparisons may be influenced by differences in age and sex composition across survey years, particularly in preschool populations with rapid age-related growth. Third, evidence remains limited regarding whether overweight and obesity patterns differed by sex and refined half-year age groups among preschool children. Analyses based on repeated health examination records may help reduce some of these limitations by accounting for within-child repeated measurements and demographic composition.

To fill these gaps, we conducted a five-year retrospective repeated-measures analysis using annual routine health examination records of preschool children in Wuhua District, Kunming City, Yunnan Province, Southwest China, from 2018 to 2022. The full dataset was used to describe annual patterns in anthropometric indicators and overweight and obesity prevalence. In addition, children with at least two annual health examination records were included in generalized linear mixed models to examine annual differences in overweight, obesity, and combined overweight and obesity while accounting for repeated measurements and age- and sex-composition differences. This study aimed to characterize five-year trends in overweight and obesity before and during the COVID-19 period and to identify sex- and age-specific patterns among preschool children.

## Methods

### Study design and data source

This study was a retrospective repeated-measures analysis based on annual routine health examination records of preschool children aged 3.0 to 7.0 (exclusive) years from all public and private kindergartens in Wuhua District, Kunming City, Yunnan Province, Southwest China from 2018 to 2022, employing a convenience-sampling strategy. All research data were from the Wuhua District Maternal and Child Health Care Hospital. The full analytic dataset was used to describe annual patterns in anthropometric indicators and overweight/obesity prevalence. In addition, because unique child identifiers were available in the original health examination database, repeated records from the same child across different years were linked. Children with at least two annual health examination records were included in the generalized linear mixed model (GLMM) analyses.

We adopted a methodological approach similar to that described in Wen’s study (30) by focusing on the first month of kindergarten reopening in Kunming, to reduce potential interference from other confounding factors, particularly the normalization of daily lives among young children following the lifting of restrictions, and to capture the immediate state of children returning to a collective kindergarten environment for the first time just after the end of home isolation. Specifically, annual physical examinations were conducted during the same calendar period each year, from May 25 to June 30, to improve comparability across survey years. In 2020, this period corresponded to the initial kindergarten reopening period in Kunming after COVID-19-related school closures. This timeframe was selected because kindergartens in Kunming gradually and orderly reopened from May 25, 2020 (34), and the summer vacation, during which schools are closed and no routine physical examinations are scheduled, started on July 1.

### Participants

Children aged 3.0 to 7.0 (exclusive) years from public and private kindergartens in Wuhua District were eligible for inclusion.

Inclusion criteria: (1) Children aged 3.0 to 7.0 (exclusive) years at the time of examination; (2) complete physical examination records; (3) no documented history of congenital or chronic disorders that could affect growth trajectories.

Exclusion criteria: (1) Missing data for key variables, such as sex, date of birth (DOB), date of examination (DOE), height, or weight; (2) implausible anthropometric values, e.g., height = 999 cm, weight = 0 kg; (3) Duplicate records for the same individual at the same examination time.

**Figure 1:**
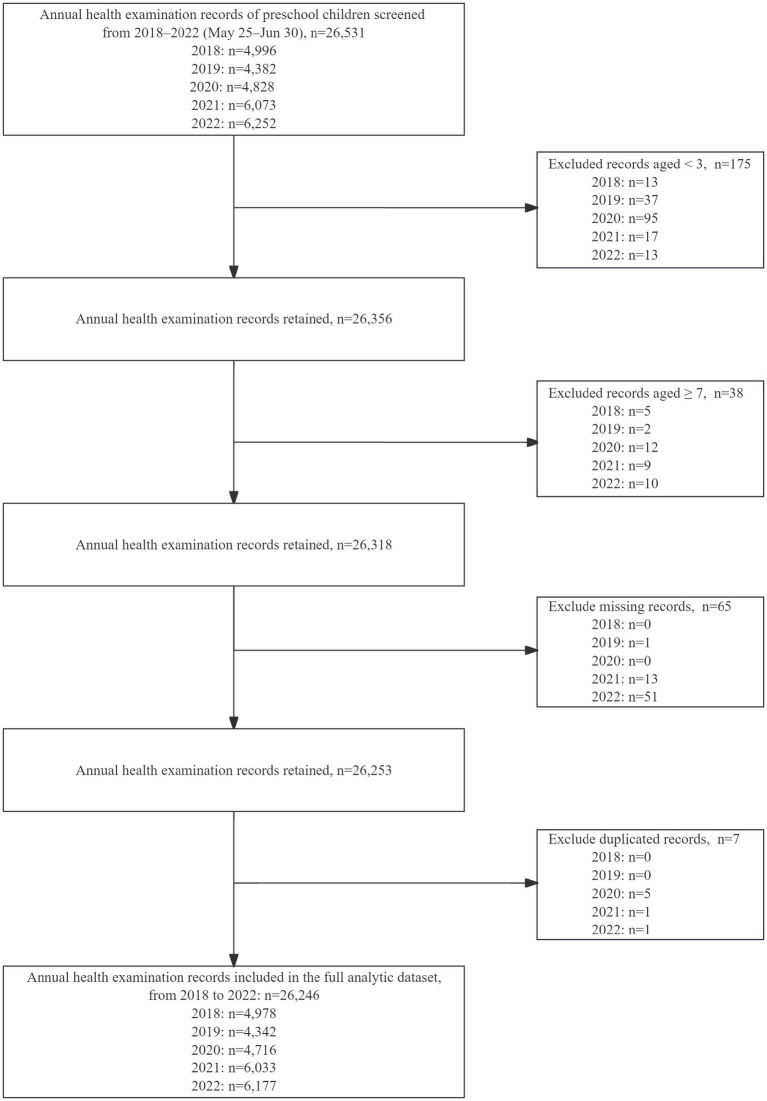
Flowchart of annual health examination records included in the full analytic dataset. The final full analytic dataset comprised 17,561 children contributing 26,246 annual health examination records from 2018 to 2022. In the flowchart, n indicates the number of annual health examination records, not unique children.

A total of 26,531 annual health examination records from 17,783 children were initially screened from 2018 to 2022, including 4,996 records in 2018, 4,382 in 2019, 4,828 in 2020, 6,073 in 2021, and 6,252 in 2022. After screening, 26,246 records from 17,561 children were included in the full analytic dataset, including 4,978 records in 2018, 4,342 in 2019, 4,716 in 2020, 6,033 in 2021, and 6,177 in 2022, respectively, and the participant selection flowchart is shown in Figure 1. Furthermore, 7,175 children with at least two annual health examination records were included, contributing 15,860 repeated records for the GLMM analyses.

### Anthropometric measurements

All anthropometric measurements were performed by hospital nurses who had completed standardized training at the Wuhua District Maternal and Child Health Care Hospital. Height was measured to the nearest 0.1 cm using a vertical stadiometer (HM1000-SZ, Jiangsu Hemei Electrical Technology Co., Ltd., Jiangsu, China), with each child standing shoeless and hatless, heels together, looking forward, and with the occiput, scapulae, buttocks, and calcanei touching the vertical column. Weight was assessed to the nearest 0.1 kg using an electronic platform scale (M303, TScale Electronics Mfg. (Kunshan) Co., Ltd., Jiangsu, China), with children standing barefoot and motionless in the center of the scale and wearing light indoor clothing, without external support.

### Variables

#### Demographic characteristics

Demographic data, including DOB, DOE, and sex, were extracted from the health examination records. Age was calculated directly according to DOE and DOB. For descriptive analyses, age was categorized into eight 6-month groups (31), including 3.0-, 3.5-, 4.0-, 4.5-, 5.0-, 5.5-, 6.0-, and 6.5- years. In the GLMM analyses, age was entered into the model as a continuous covariate (precise to 0.1 years) to preserve individual-level age variation and reduce model complexity.

#### Anthropometric data

Weight and height values were also obtained directly from the hospital’s electronic records. Body-mass index (BMI) was calculated according to the formula: BMI (kg/m^2^) = weight (kg)/height^2^ (m^2^), and was rounded to two decimal places.

#### Overweight and obesity

Overweight and obesity were classified according to the latest national standard—*Growth Standard for Children Under 7 Years of Age*, issued by the National Health Commission of China on September 19, 2022 (35). Nutritional status was evaluated using age-adjusted BMI z-scores with standard deviation (SD) cutoffs. Overweight was defined as +1 SD ≤ BMI-for-age < +2 SD, and obesity was defined as BMI-for-age ≥ + 2 SD. The 2022 standard was applied uniformly to all survey years to ensure internal comparability of overweight and obesity classification across the study period. Combined overweight and obesity was abbreviated as OwO.

### Statistical analysis

Numerical variables, including weight, height, and BMI, were described as the mean and standard deviation (M ± SD) and analyzed utilizing one-way analysis of variance (ANOVA). Post-hoc pairwise comparisons were performed using Tukey’s HSD test.

Categorical variables, including overweight, obesity and OwO status, were shown as frequency (n) and proportion (%). The chi-square test or Fisher’s exact probability test was used to assess differences among independent sample groups. Post-hoc pairwise comparisons were adjusted using the Bonferroni correction.

GLMM analyses were performed using the repeated-measures subset of children with at least two annual health examination records (31). Overweight, obesity, and OwO status were modeled as binary outcomes, respectively. Survey year was included as the main fixed effect, with 2020 used as the reference year. In the overall models, sex and continuous age at examination were included as fixed effects. In sex-stratified models, continuous age at examination was included as a fixed effect. Adjusted odds ratios (*OR*), 95% confidence intervals (CI), and *p* values were reported.

Statistical analyses were conducted using SPSS software, version 25.0 (IBM), R (v4.2.2; R Foundation), and the Free Statistics platform (v2.0^1^), a Python-based GUI with an R backend. A two-tailed *p* value < 0.05 was considered statistically significant. Figures were generated using GraphPad Prism 10.5.0.

### Ethics statement

This study was approved by the Medical Ethics Committee of the Affiliated Hospital of Chengdu University (Approval No. PJ2023-019-03). All data were collected anonymously to ensure participant confidentiality.

## Results

### Demographic characteristics

A total of 26,246 annual health examination records from 17,561 preschool children were included from 2018 to 2022. Of these records, 4,978 (19.0%) were from 2018, 4,342 (16.5%) from 2019, 4,716 (18.0%) from 2020, 6,033 (23.0%) from 2021, and 6,177 (23.5%) from 2022, respectively. Boys accounted for 13,542 records (51.6%) and girls for 12,704 records (48.4%). The annual number of records increased from 2,563 in 2018 to 3,197 in 2022 among boys and from 2,415 in 2018 to 2,980 in 2022 among girls (Supplementary Table 1).

The age range covered children aged 3.0 to < 7.0 years, and the age distribution differed across survey years. In most years, the majority of records were concentrated in the 4.0–5.5-year age groups. Notably, the 5.5-year age group comprised the largest single-year subgroup in 2022 (n = 1,307). In contrast, the number of records in the 3.0-year age group was markedly lower in 2021 (n = 92) and 2022 (n = 79). Furthermore, the age distribution pattern was similar between boys and girls. It is noteworthy that the absolute number of boys’ records was slightly higher than that of girls’ across multiple age groups and years (Supplementary Table 1).

### Descriptive anthropometric characteristics

**Figure 2:**
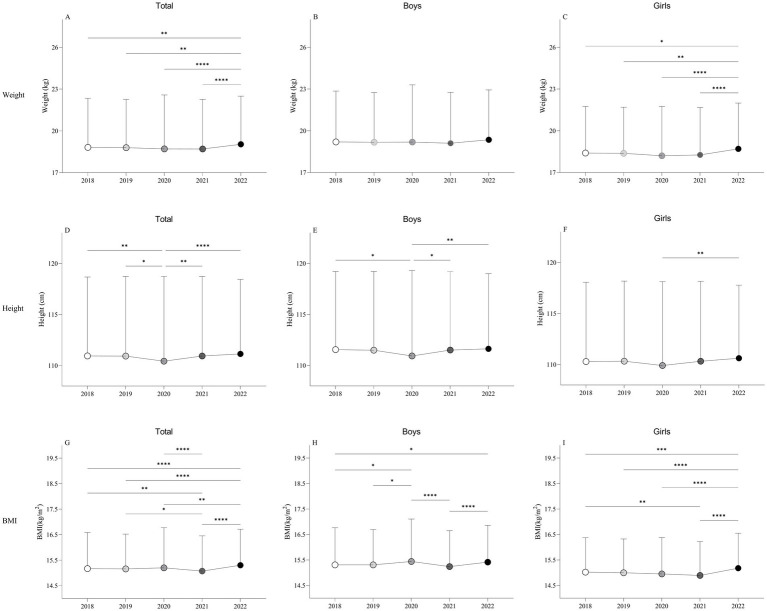
Descriptive annual patterns of height, weight, and BMI in the full analytic dataset. **(A)** Annual weight trends for the total population; **(B)** annual weight trends for boys; **(C)** annual weight trends for girls; **(D)** annual height trends for the total population; **(E)** annual height trends for boys; **(F)** annual height trends for girls; **(G)** annual BMI trends for the total population; (H) annual BMI trends for boys; and (I) annual BMI trends for girls. The full analytic dataset included 17,561 children and 26,246 annual health examination records. Points indicate mean values, and error bars indicate standard deviations. Pairwise comparisons were performed using Tukey’s HSD test. *p < 0.05, **p < 0.01, ***p < 0.001, ****p < 0.0001. BMI: body mass index.

Height, weight, and BMI were summarized as descriptive anthropometric indicators before the analysis of overweight, obesity and OwO. In the full analytic dataset, the trends of crude mean height and weight were consistent, showing an initial decrease followed by an increase, with the lowest values observed in 2020 at (110.43 ± 8.32) cm and (18.70 ± 3.88) kg, respectively. In contrast, crude mean BMI exhibited a fluctuating pattern, increasing slightly from 2019 (15.16 ± 1.36 kg/m^2^) to 2020 (15.20 ± 1.57 kg/m^2^), followed by a decline to its lowest point in 2021 (15.07 ± 1.38 kg/m^2^) and subsequently increased to the highest value in 2022 (15.30 ± 1.41 kg/m^2^). One-way ANOVA revealed statistically significant annual variations in height, weight, and BMI across the 5 years, with *F* values of 6.019, 8.725, and 20.035, respectively (all *p* values <0.001) (Figure 2 and Table 1).

**Table 1:** Overall and sex-specific annual anthropometric characteristics in the full analytic dataset, 2018–2022.

Year	Total (*n* = 26,246)				Boys (*n* = 13,542)				Girls (*n* = 12,704)			
	*n*	Height (cm)	Weight (kg)	BMI (kg/m^2^)	*n*	Height (cm)	Weight (kg)	BMI (kg/m^2^)	*n*	Height (cm)	Weight (kg)	BMI (kg/m^2^)
Total	26,246	110.90 ± 7.77	18.82 ± 3.58	15.18 ± 1.43	13,542	111.44 ± 7.74	19.21 ± 3.71	15.34 ± 1.47	12,704	110.32 ± 7.75	18.40 ± 3.38	15.01 ± 1.36
2018	4,978	110.95 ± 7.73	18.81 ± 3.53	15.17 ± 1.41	2,563	111.56 ± 7.65	19.20 ± 3.65	15.31 ± 1.46	2,415	110.30 ± 7.77	18.40 ± 3.35	15.02 ± 1.35
2019	4,342	110.94 ± 7.80	18.79 ± 3.47	15.16 ± 1.36	2,248	111.50 ± 7.70	19.18 ± 3.57	15.31 ± 1.38	2,094	110.33 ± 7.85	18.38 ± 3.32	15.00 ± 1.33
2020	4,716	110.43 ± 8.32	18.70 ± 3.88	15.20 ± 1.57	2,400	110.93 ± 8.39	19.19 ± 4.12	15.44 ± 1.67	2,316	109.91 ± 8.22	18.20 ± 3.54	14.95 ± 1.43
2021	6,033	110.94 ± 7.79	18.70 ± 3.56	15.07 ± 1.38	3,134	111.51 ± 7.70	19.10 ± 3.66	15.24 ± 1.41	2,899	110.32 ± 7.83	18.26 ± 3.40	14.89 ± 1.33
2022	6,177	111.15 ± 7.29	19.04 ± 3.46	15.30 ± 1.41	3,197	111.64 ± 7.37	19.35 ± 3.58	15.42 ± 1.44	2,980	110.63 ± 7.16	18.70 ± 3.29	15.18 ± 1.37
*F* value		6.019	8.725	20.035		3.345	1.915	9.015		2.792	8.985	17.965
*p* value		<0.001	<0.001	<0.001		0.010	0.105	<0.001		0.025	<0.001	<0.001

The full analytic dataset included 17,561 children and 26,246 annual health examination records, including 13,542 records from boys and 12,704 records from girls. n: the number of annual health examination records; BMI: body mass index. *F* values and *p-*values were derived from one-way ANOVA test for comparison across five survey years within total sample and each sex group. *P* < 0.05 was considered statistically significant.

These crude anthropometric findings were interpreted descriptively because annual mean values may be influenced by differences in age composition across survey years. Detailed sex- and age-stratified results for height, weight, and BMI are shown in Figure 2, Table 1, and Supplementary Table 2. The following analyses therefore focused on overweight, obesity, and OwO, which were further evaluated using both descriptive comparisons and GLMM analyses.

### Comparison of overweight, obesity and OwO

#### Total comparison

**Figure 3:**
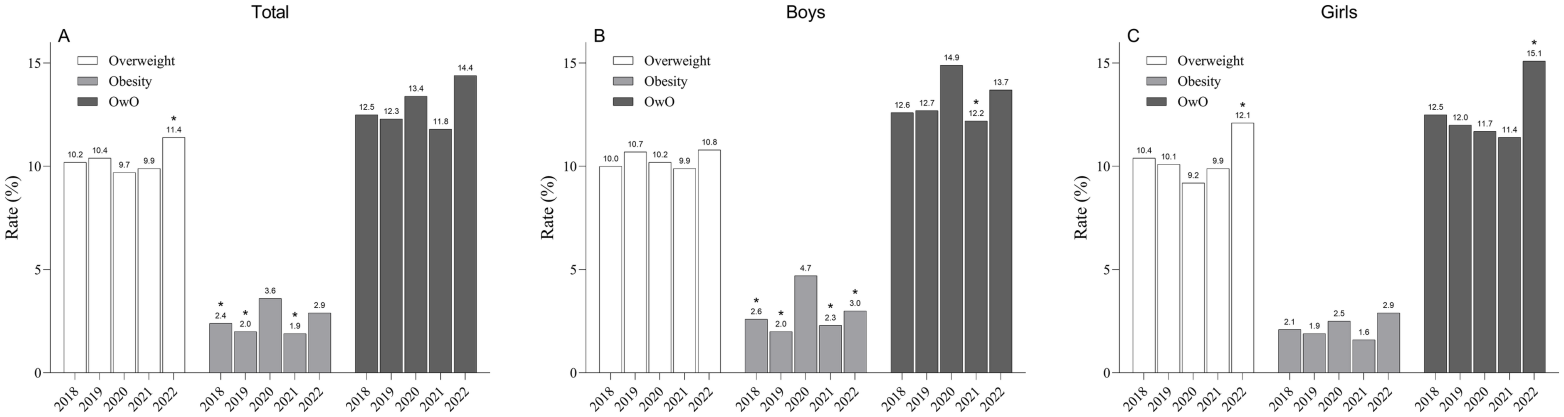
Crude prevalence of overweight, obesity, and OwO in the full analytic dataset. **(A)** Prevalence trends for the total population; **(B)** prevalence trends for boys; and **(C)** prevalence trends for girls. The full analytic dataset included 17,561 children and 26,246 annual health examination records. Prevalence estimates are unadjusted. *p < 0.0125, compared with 2020 after Bonferroni correction. OwO: combined overweight and obesity.

In the full analytic dataset of 26,246 health examination records from 17,561 children, the overall prevalence of OwO fluctuated between 11.8 and 14.4% across the five survey years. The overweight prevalence reached its highest level in 2022 (11.4%), whereas the obesity rate exhibited a distinct peak in 2020 (3.6%) (Figure 3A). Trend analysis indicated that no significant linear temporal trends were observed for overweight (Mantel–Haenszel *χ^2^* = 2.824, *p* = 0.093) or obesity (Mantel–Haenszel *χ^2^* = 2.354, *p* = 0.125). In contrast, OwO showed a modest but statistically significant upward trend over the study period (Mantel–Haenszel *χ^2^* = 5.058, *p* = 0.025), despite interannual fluctuations. The chi-square test showed statistically significant annual differences for overweight (*χ^2^* = 11.277, *p* = 0.024), obesity (*χ^2^* = 42.248, *p* < 0.001), and OwO (*χ^2^* = 21.088, *p* < 0.001) (Table 2).

**Table 2:** Overall and sex-specific prevalence of overweight, obesity, and OwO in the full analytic dataset, 2018–2022.

Year	Total (*n* = 26,246)				Boys (*n* = 13,542)				Girls (*n* = 12,704)			
	*n*	Overweight, *n* (%)	Obesity, *n* (%)	OwO, *n* (%)	*n*	Overweight, *n* (%)	Obesity, *n* (%)	OwO, *n* (%)	*n*	Overweight, *n* (%)	Obesity, *n* (%)	OwO, *n* (%)
Total	26,246	2,719 (10.4)	671 (2.6)	3,390 (12.9)	13,542	1,395 (10.3)	390 (2.9)	1,785 (13.2)	12,704	1,324 (10.4)	281 (2.2)	1,605 (12.6)
2018	4,978	507 (10.2)	117 (2.4) *	624 (12.5)	2,563	256 (10.0)	66 (2.6) *	322 (12.6)	2,415	251 (10.4)	51 (2.1)	302 (12.5)
2019	4,342	451 (10.4)	85 (2.0) *	536 (12.3)	2,248	240 (10.7)	45 (2.0) *	285 (12.7)	2,094	211 (10.1)	40 (1.9)	251 (12.0)
2020	4,716	459 (9.7)	171 (3.6)	630 (13.4)	2,400	245 (10.2)	113 (4.7)	358 (14.9)	2,316	214 (9.2)	58 (2.5)	272 (11.7)
2021	6,033	596 (9.9)	116 (1.9) *	712 (11.8)	3,134	310 (9.9)	71 (2.3) *	381 (12.2) *	2,899	286 (9.9)	45 (1.6)	331 (11.4)
2022	6,177	706 (11.4) *	182 (2.9)	888 (14.4)	3,197	344 (10.8)	95 (3.0) *	439 (13.7)	2,980	362 (12.1) *	87 (2.9)	449 (15.1) *
*χ^2^* value		11.277	42.248	21.088		1.934	40.061	11.388		14.203	14.638	22.361
*P* value		0.024	<0.001	<0.001		0.748	<0.001	0.023		0.007	0.006	<0.001
Trend *χ^2^* value		2.824	2.354	5.058		0.224	0.614	0.661		3.705	2.058	5.785
*P* for trend		0.093	0.125	0.025		0.636	0.433	0.416		0.054	0.151	0.016

OwO: combined overweight & obesity; n: number of health examination records. The full analytic dataset included 17,561 children and 26,246 annual health examination records, including 13,542 records from boys and 12,704 records from girls. Overall p values are unadjusted and were derived from Pearson’s chi-square test for between-year comparisons within the total sample and each sex group. P for trend was calculated using the Mantel–Haenszel test for trend. *p < 0.0125, compared with 2020 after Bonferroni correction for pre-specified pairwise comparisons.

Using 2020 as the reference year, Bonferroni-corrected pairwise comparisons showed that overweight prevalence in 2022 was significantly higher than that in 2020 (11.4% vs. 9.7%, *p* < 0.0125), whereas obesity prevalence in 2018, 2019, and 2021 was significantly lower than that in 2020 (2.4, 2.0, and 1.9%, respectively, vs. 3.6%; all *p* < 0.0125). No Bonferroni-corrected pairwise difference in OwO prevalence was observed between 2020 and the other years (Figure 3A and Table 2).

#### Sex-stratified comparison

Sex-stratified analyses showed different patterns between boys and girls. Among boys, obesity prevalence reached a peak in 2020 (4.7%), and significant interannual differences were observed for obesity (*χ^2^* = 40.061, *p* < 0.001) and OwO (*χ^2^* = 11.388, *p* = 0.023), but not for overweight (*χ^2^* = 1.934, *p* = 0.748) (Table 2). In Bonferroni-corrected comparisons, obesity prevalence in 2020 was significantly higher than in the other survey years (4.7% vs. 2.6, 2.0, 2.3, and 3.0%, respectively; all *p* < 0.0125). In addition, OwO rate in 2020 was also significantly higher than that in 2021 (14.9% vs. 12.2%, *p* < 0.0125) (Figure 3B and Table 2).

Among girls, significant annual differences were detected for overweight (*χ^2^* = 14.203, *p* = 0.007), obesity (*χ^2^* = 14.638, *p* = 0.006), and OwO (*χ^2^* = 22.361, *p* < 0.001) (Table 2). Furthermore, the prevalence of overweight (12.1% vs. 9.2%, *p* < 0.0125) and OwO (15.1% vs. 11.7%, *p* < 0.0125) in 2022 was significantly higher than that in 2020, and OwO showed a significant upward trend across the 5 years (Figure 3C and Table 2).

#### Sex- and age-stratified comparison

In sex- and age-stratified analyses, most age groups did not show significant annual differences in overweight, obesity, or OwO prevalence. Significant differences among boys were mainly observed for obesity in the older age groups, particularly the 5.5-, 6.0-, and 6.5-year groups. Among girls, significant differences were mainly observed in the 4.0- and 4.5-year groups, particularly for obesity or OwO (Supplementary Table 3).

### Repeated-measures subset and GLMM analysis

#### Crude prevalence in the repeated-measures subset

A total of 7,175 children with at least two annual health examination records were included in the repeated-measures subset, contributing 15,860 of the 26,246 records. Of these records, 8,201 (51.7%) were from boys and 7,659 (48.3%) from girls. Before GLMM model fitting, crude rates of overweight, obesity, and OwO in this subset were summarized.

**Figure 4:**
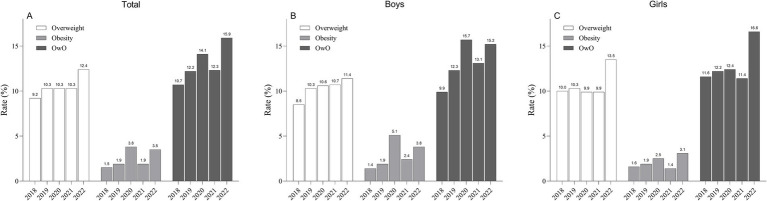
Crude prevalence of overweight, obesity, and OwO in the repeated-measures subset for GLMM analyses. **(A)** Prevalence trends for the total population; **(B)** prevalence trends for boys; and **(C)** prevalence trends for girls. The repeated-measures subset included 7,175 children with at least two annual health examination records, contributing 15,860 records. Prevalence estimates are unadjusted. OwO: combined overweight and obesity; GLMM: generalized linear mixed model.

In the total repeated-measures subset, overweight prevalence was relatively stable from 2019 to 2021 and was highest in 2022 (12.4%). Obesity prevalence was highest in 2020 (3.8%) and remained relatively high in 2022 (3.5%). The crude prevalence of OwO increased from 10.7% in 2018 to 15.9% in 2022, with higher values observed in 2020 and 2022 (Figure 4A).

Sex-stratified patterns differed. Among boys, obesity prevalence peaked in 2020 (5.1%), while OwO was also highest in 2020 (15.7%) and remained high in 2022 (15.2%) (Figure 4B). Among girls, overweight, obesity, and OwO were all highest in 2022, reaching 13.5, 3.1, and 16.6%, respectively (Figure 4C). These unadjusted patterns were further evaluated using GLMM to account for within-child repeated measurements and covariates.

#### Total GLMM analysis

Overweight, obesity and OwO status were modeled as binary outcomes, respectively, with 2020 as the reference year. In the overall models adjusted for sex and age, the odds of overweight were significantly higher only in 2022 than in 2020 (*OR* = 1.92; 95% CI: 1.26–2.92, *p* = 0.002), whereas no significant differences were observed for 2018, 2019, or 2021 (Table 3).

**Table 3:** GLMM analysis of overweight, obesity, and OwO in the repeated-measures subset (*N* = 7,175, *n* = 15,860).

Variables		Overweight		Obesity		OwO	
		O*R* (95% CI)	*P-*value	*OR* (95% CI)	*P-*value	*OR* (95% CI)	*P-*value
Total	2020	1.0 (ref)		1.0 (ref)		1.0 (ref)	
	2018	0.84 (0.50–1.41)	0.508	0.02 (<0.01–0.20)	0.001	0.37 (0.19–0.74)	0.004
	2019	1.20 (0.81–1.79)	0.363	0.02 (<0.01–0.09)	<0.001	0.61 (0.38–0.97)	0.037
	2021	1.27 (0.86–1.86)	0.228	0.03 (0.01–0.08)	<0.001	0.57 (0.37–0.89)	0.012
	2022	1.92 (1.26–2.92)	0.002	0.91 (0.33–2.49)	0.858	1.82 (1.14–2.89)	0.012
Boys	2020	1.0 (ref)		1.0 (ref)		1.0 (ref)	
	2018	0.74 (0.36–1.52)	0.413	<0.01 (<0.01–0.01)	<0.001	0.12 (0.04–0.35)	<0.001
	2019	1.34 (0.78–2.30)	0.290	<0.01 (<0.01–0.01)	<0.001	0.34 (0.17–0.67)	0.002
	2021	1.73 (1.03–2.91)	0.038	0.02 (<0.01–0.07)	<0.001	0.64 (0.36–1.14)	0.127
	2022	1.76 (0.98–3.13)	0.057	0.47 (0.13–1.69)	0.250	1.12 (0.59–2.12)	0.729
Girls	2020	1.0 (ref)		1.0 (ref)		1.0 (ref)	
	2018	0.93 (0.44–2.00)	0.860	2.61 (0.20–34.57)	0.467	0.81 (0.32–2.03)	0.647
	2019	1.05 (0.58–1.89)	0.878	1.29 (0.21–7.72)	0.783	0.80 (0.41–1.55)	0.509
	2021	0.83 (0.47–1.48)	0.534	0.09 (0.02–0.47)	0.004	0.30 (0.16–0.59)	<0.001
	2022	2.06 (1.11–3.82)	0.022	8.02 (1.12–57.53)	0.038	2.07 (1.05–4.09)	0.035

GLMM: Generalized linear mixed model; OwO: combined overweight & obesity; N: the number of children with at least two annual health examination records; n: number of health examination records; OR: odds ratio, CI: confidence interval. The repeated-measures subset included 7,175 children with at least two annual health examination records, contributing 15,860 records. Year of examination was included as the main fixed effect, with 2020 as the reference year. Child ID was included as a random effect to account for within-child correlation across repeated annual measurements. Continuous age at examination and sex were adjusted in the overall model; continuous age at examination was adjusted in sex-stratified models. A two-sided *P* value < 0.05 was considered statistically significant.

In contrast, the odds of obesity were significantly lower in 2018 (*OR* = 0.02; 95% CI: < 0.01–0.20; *p* = 0.001), 2019 (*OR* = 0.02, 95% CI = < 0.01–0.09, *p* < 0.001), and 2021 (*OR* = 0.03, 95% CI = 0.01–0.08, *p* < 0.001) than in 2020, but no significant difference was observed between 2022 and 2020 (*p* = 0.858). Furthermore, the odds of OwO were significantly lower in 2018 (*OR* = 0.37, 95% CI: 0.19–0.74, *p* = 0.004), 2019 (*OR* = 0.61, 95% CI: 0.38–0.97, *p* = 0.037), and 2021 (*OR* = 0.57, 95% CI: 0.37–0.89, *p* = 0.012) than in 2020, but were significantly higher in 2022 (*OR* = 1.82, 95% CI: 1.14–2.89, *p* = 0.012) (Table 3).

#### Subgroup GLMM analysis by sex

In sex-stratified models, age was adjusted as a covariate. Among boys, the odds of overweight were significantly higher in 2021 (*OR* = 1.73, 95% CI: 1.03–2.91, *p* = 0.038) than in 2020. The odds were also higher in 2022, but the difference did not reach statistical significance (*OR* = 1.76, 95% CI: 0.98–3.13, *p* = 0.057). Unlike overweight, the odds of obesity were significantly lower in 2018, 2019, and 2021 than in 2020, whereas no significant difference was observed between 2022 and 2020 (*p* = 0.250). In addition, the odds of OwO were significantly lower in 2018 (*OR* = 0.12, 95% CI: 0.04–0.35, *p* < 0.001) and 2019 (*OR* = 0.34, 95% CI: 0.17–0.67, *p* = 0.002) than in 2020, but no significant differences were observed in 2021 (*OR* = 0.64, *p* = 0.127) or 2022 (*OR* = 1.12, *p* = 0.729) (Table 3).

Among girls, the odds of overweight were significantly higher only in 2022 (*OR* = 2.06, 95% CI: 1.11–3.82, *p* = 0.022), with no significant differences observed for 2018, 2019, or 2021. Moreover, the odds of obesity were significantly lower in 2021 than in 2020 (*OR* = 0.09, 95% CI: 0.02–0.47, *p* = 0.004), whereas the estimate was significantly higher in 2022 (*OR* = 8.02, 95% CI: 1.12–57.53, *p* = 0.038). No significant differences were found for 2018 or 2019. A similar pattern was observed for OwO, with significantly lower odds in 2021 than in 2020 (*OR* = 0.30, 95% CI: 0.16–0.59, *p* < 0.001) and significantly higher odds in 2022 (*OR* = 2.07, 95% CI: 1.05–4.09, *p* = 0.035). No significant differences were identified for 2018 or 2019 (Table 3).

## Discussion

In this 5-year retrospective repeated-measures analysis of annual health examination records from preschool children in Southwest China, we found distinct temporal and sex-specific patterns in overweight, obesity, and OwO before and during the COVID-19 period. In the full analytic dataset, obesity prevalence was highest in 2020, whereas overweight and OwO prevalence were highest in 2022. These descriptive patterns were broadly consistent with the findings from GLMM analyses based on the repeated-measures subset. With 2020 as the reference year, the adjusted odds of obesity were lower in 2018, 2019, and 2021 than in 2020, while the adjusted odds of overweight and OwO were higher in 2022 than in 2020. Sex-stratified GLMM analyses suggested that the elevated obesity burden around 2020 was more evident among boys, whereas the increase in overweight, obesity, and OwO in 2022 was more apparent among girls; the obesity estimate among girls in 2022 also increased, but its wide confidence interval indicates substantial uncertainty. These findings indicate that the temporal pattern of preschool overweight and obesity during the COVID-19 period was heterogeneous by sex and did not follow a simple uniform trend.

Our findings are partly consistent with the study by Long et al. (31), which used annual health examination data from kindergarten children aged 3–7 years in Jiading District, Shanghai, from 2018 to 2021. Similar to our analytic strategy, Long et al. first described annual patterns using all available health examination records and then conducted GLMM analyses using a repeated data subset of children with at least two annual examination records. They reported that both overweight and obesity were highest in 2020 and that, compared with 2020, the adjusted odds of overweight and obesity were lower in 2018, 2019, and 2021. In our study, the obesity pattern was similar: the adjusted odds of obesity were lower in 2018, 2019, and 2021 than in 2020, supporting the interpretation that the initial kindergarten reopening period after COVID-19-related closures was associated with a higher obesity burden among preschool children.

However, our findings also extend those of Long et al. in three ways. First, our observation window included 2022, allowing us to assess later patterns during the subsequent COVID-19 control period. Unlike Long et al., whose data ended in 2021, we observed that the adjusted odds of overweight and OwO were higher in 2022 than in 2020, especially among girls. Second, our GLMM included child ID as a random effect to directly account for within-child correlation across repeated annual measurements. Third, by combining the full analytic dataset with an individual-level repeated-measures subset, our study provided both population-level descriptive trends and adjusted repeated-measures estimates. These findings suggest that the higher obesity burden around 2020 among boys and the later increase in overweight and OwO among girls may represent different phases of weight-status change during the COVID-19 period.

Our results also partly align with Wen et al. (30), who reported that obesity prevalence among Chinese preschool children increased after COVID-19-related school closures, although overweight prevalence decreased in their study. This pattern is consistent with our observation that obesity was more prominent around 2020, but differs from our findings that overweight and OwO were higher in 2022. The discrepancy may be related to differences in study regions, age ranges, measurement windows, and analytic approaches. Wen et al. compared anthropometric changes before and after the school closure period, with post-closure measurements obtained in August to September 2020. In contrast, our study used annual health examination records from 2018 to 2022 and included a GLMM analysis based on repeated records, which enabled us to examine both the initial reopening period in 2020 and the later control period in 2021–2022.

A recent study from the same district in Kunming provides additional local evidence. Cai et al. (36) followed 1,298 preschool children with complete annual health examination records from 2018 to 2021 and reported increases in overweight and obesity during the lockdown period. Their paired-cohort design strengthened internal comparability by tracking the same children across four consecutive years. In contrast, our study included a larger full analytic dataset and a larger repeated-measures subset extending the observation period to 2022. Together, these two studies suggest that overweight and obesity among preschool children in Wuhua District increased during and after the COVID-19-related disruption, although the specific timing and sex-specific patterns differed according to study design, inclusion criteria, and analytic methods.

Although our routine health examination records did not include lifestyle variables, previous longitudinal evidence from Chinese children provides a plausible behavioral context. He et al. (37) reported that during the COVID-19 pandemic, overweight and obesity prevalence increased, while daily physical activity and sleep duration decreased and screen time increased. Their generalized estimating equations (GEE) model analysis further suggested that lower physical activity, shorter sleep duration, and longer screen time were associated with obesity. These findings support the possibility that disruptions to kindergarten routines, reduced outdoor activity, increased sedentary behavior, altered sleep, and changes in dietary behaviors may have contributed to the temporal patterns observed in our study. However, because these behaviors were not directly measured in our dataset, these mechanisms should be interpreted as plausible explanations rather than confirmed pathways.

Our study found a sex-specific temporal pattern in overweight, obesity and OwO, characterized by an earlier obesity increase among boys and a later simultaneous increase in overweight, obesity and OwO among girls. This finding aligns with the concerns raised by Hong et al. (38) that the COVID-19 pandemic might reverse the slowing trend in childhood obesity. Among boys, the higher obesity burden around 2020 may be related to abrupt reductions in physical activity during the closure and early reopening period. Boys generally have higher baseline physical activity levels and may be more prone to increased screen time (39); therefore, restrictions on outdoor play and structured kindergarten activities may have affected their energy balance more immediately. Lockdown-related changes in dietary behaviors, including increased consumption of energy-dense and ultra-processed foods, and reduced fruit and vegetable intake, may have further contributed to this pattern (40, 41).

In contrast, to our knowledge, few studies have reported a simultaneous increase in overweight, obesity, and OwO among girls in the later COVID-19 control period. This may reflect a later-emerging pattern rather than an immediate change. The behavioral inertia hypothesis (42) posits that girls may have greater difficulty recovering from unhealthy lifestyle habits formed during the pandemic. Previous studies have shown that the COVID-19 pandemic was associated with reduced physical activity, increased sedentary behavior and screen time, altered sleep patterns, and changes in dietary behaviors among children (37, 40–43). Longitudinal evidence from Chinese children further suggests that reduced physical activity, shorter sleep duration, and longer screen time were associated with obesity during the pandemic (37). In addition, early childhood is a sensitive period for adiposity rebound, and accelerated BMI gain during this period is associated with sustained obesity risk later in childhood (44, 45). Environmental disruptions during this sensitive period may exert more lasting effects on body fat trajectories. Psychosocial factors (43) suggest that girls may be more vulnerable to stress-related emotional eating due to pandemic-associated pressures. Indirect support for this hypothesis is provided by Huang et al. (46), who found that a positive psychological and behavioral state was a protective factor against excessive weight gain. Nevertheless, these interpretations remain hypothetical because individual-level behavioral, dietary, sleep, and family socioeconomic data were unavailable in our dataset.

### Limitations

Several limitations should also be acknowledged. First, this was an observational study based on routinely collected health examination records; therefore, causal effects of COVID-19-related school closures or control policies cannot be inferred. Second, although GLMM analyses accounted for repeated measurements and adjusted for age and sex, residual confounding may remain. The dataset did not include individual-level lifestyle data, including physical activity, dietary intake, screen time, sleep patterns, sedentary behavior, parental age and education, household socioeconomic status, or family environment (e.g., dietary habits, sleep patterns, and exercise levels). Third, routine health examination participation varied across years, and differences in sample composition may have influenced crude annual prevalence estimates. However, the repeated-measures GLMM analysis partly reduced this concern by including children with at least two annual health examination records and accounting for within-child correlation. Fourth, this was a single-center study, conducted in one urban district in Southwest China, and the findings may not be generalizable to all preschool children in China or other countries with different socioeconomic and cultural backgrounds. Fifth, overweight and obesity were classified using BMI-based criteria, which do not directly measure body fat distribution or body composition. Finally, the relatively small number of obesity cases may have affected the stability of some GLMM estimates, particularly in sex-stratified analyses. Some obesity-related estimates had wide confidence intervals, and these results should therefore be interpreted cautiously.

## Conclusion

This five-year retrospective repeated-measures analysis identified sex-specific temporal patterns in overweight, obesity, and OwO among preschool children before and during the COVID-19 period in Southwest China. The obesity burden was higher around the initial kindergarten reopening period in 2020, particularly among boys, whereas overweight, obesity and OwO were higher in 2022, particularly among girls. These findings suggest that changes in preschool weight status during the COVID-19 period were heterogeneous by sex and survey year. Continued monitoring of preschool children’s weight status and targeted early-life obesity prevention strategies are warranted after major public health disruptions.

## Data Availability

The raw data supporting the conclusions of this article will be made available by the authors, without undue reservation.

## Footnotes

1
http://www.clinicalscientists.cn/freestatistics
