# Supplementary material for: Five-year trends in overweight and obesity among preschool children before and during the COVID-19 period: a retrospective repeated-measures analysis in Southwest China
Source: Front Public Health. 2026 Jul 17;14:1842094. doi: 10.3389/fpubh.2026.1842094 (PMC13424550; doi:10.3389/fpubh.2026.1842094)
Supplement: Supplementary file 1 [file Table_1.DOCX]

**Supplementary Tables**

Table 1 Demographic characteristics of annual health examination records among preschool children in Wuhua District from 2018 to 2022 (n=26,246), n (%)

| Age (yrs) | Total (n=26,246) | | | | | Boy (n=13,542) | | | | | Girl (n=12,704) | | | | |
| --- | --- | --- | --- | --- | --- | --- | --- | --- | --- | --- | --- | --- | --- | --- | --- |
|  | 2018 (n=4,978) | 2019 (n=4,342) | 2020 (n=4,716) | 2021 (n=6,033) | 2022 (n=6,177) | 2018 (n=2,563) | 2019 (n=2,248) | 2020 (n=2,400) | 2021 (n=3,134) | 2022 (n=3,197) | 2018 (n=2,415) | 2019 (n=2,094) | 2020 (n=2,316) | 2021 (n=2,899) | 2022 (n=2,980) |
| 3.0- | 99  (2.0) | 120  (2.8) | 232  (4.9) | 92  (1.5) | 79  (1.3) | 49  (1.9) | 55  (2.4) | 120  (5.0) | 39  (1.2) | 48  (1.5) | 50  (2.1) | 65  (3.1) | 112  (4.8) | 53  (1.8) | 31  (1.0) |
| 3.5- | 586  (11.8) | 352  (8.1) | 648  (13.7) | 564  (9.4) | 482  (7.8) | 284  (11.1) | 173  (7.7) | 326  (13.6) | 286  (9.1) | 254  (7.9) | 302  (12.5) | 179  (8.5) | 322  (13.9) | 278  (9.6) | 228  (7.7) |
| 4.0- | 673  (13.5) | 665  (15.3) | 708  (15.0) | 1,036 (17.2) | 885  (14.3) | 335  (13.1) | 366  (16.3) | 375  (15.6) | 519  (16.6) | 453  (14.2) | 338  (14.0) | 299  (14.3) | 333  (14.4) | 517  (17.8) | 432  (14.5) |
| 4.5- | 739  (14.8) | 858  (19.8) | 486  (10.3) | 1,260 (20.9) | 959  (15.5) | 398  (15.5) | 434  (19.3) | 240  (10.0) | 667  (21.3) | 471  (14.7) | 341  (14.1) | 424  (20.2) | 246  (10.6) | 593  (20.5) | 488  (16.4) |
| 5.0- | 706  (14.2) | 643  (14.8) | 581  (12.3) | 969  (16.1) | 1,123  (18.2) | 364  (14.2) | 329  (14.6) | 296  (12.3) | 518  (16.5) | 566  (17.7) | 342  (14.2) | 314  (15.0) | 285  (12.3) | 451  (15.6) | 557  (18.7) |
| 5.5- | 966  (19.4) | 661  (15.2) | 916  (19.4) | 656  (10.9) | 1,307  (21.2) | 506  (19.7) | 339  (15.1) | 459  (19.1) | 345  (11.0) | 690  (21.6) | 460  (19.0) | 322  (15.4) | 457  (19.7) | 311  (10.7) | 617  (20.7) |
| 6.0- | 845  (17.0) | 603  (13.9) | 771  (16.4) | 836  (13.9) | 1,018  (16.5) | 426  (16.6) | 318  (14.1) | 384  (16.0) | 447  (14.3) | 545  (17.0) | 419  (17.3) | 285  (13.6) | 387  (16.7) | 389  (13.4) | 473  (15.9) |
| 6.5- | 364  (7.3) | 440  (10.1) | 374  (7.9) | 620  (10.3) | 324  (5.2) | 201  (7.8) | 234  (10.4) | 200  (8.3) | 313  (10.0) | 170  (5.3) | 163  (6.7) | 206  (9.8) | 174  (7.5) | 307  (10.6) | 154  (5.2) |

n: the number of annual health examination records.

Table 2 Sex- and age-stratified comparison of height, weight, and BMI across five years in the full analytic dataset (n=26,246)

| Age | Year | Boys (n=13,542) | | | | Girls (n=12,704) | | | |
| --- | --- | --- | --- | --- | --- | --- | --- | --- | --- |
|  |  | Records, n | Height (cm) | Weight (kg) | BMI (kg/m^2^) | Records, n | Height (cm) | Weight (kg) | BMI (kg/m^2^) |
| 3.0- | Total | 311 | 97.94 ± 3.90 | 14.67 ± 1.58 | 15.27 ± 1.13 | 311 | 96.83 ± 3.83 | 14.07 ± 1.65 | 14.97 ± 1.07 |
|  | 2018 | 49 | 98.05 ± 3.80 | 14.73 ± 1.44 | 15.30 ± 0.90 | 50 | 97.03 ± 4.11 | 14.59 ± 1.74 | 15.44 ± 0.90 |
|  | 2019 | 55 | 97.18 ± 4.64 | 14.64 ± 1.97 | 15.46 ± 1.37 | 64 | 95.53 ± 4.00 | 13.59 ± 1.42 | 14.87 ± 1.08 |
|  | 2020 | 120 | 97.69 ± 3.62 | 14.50 ± 1.50 | 15.18 ± 1.19 | 113 | 96.85 ± 3.67 | 13.99 ± 1.67 | 14.88 ± 1.11 |
|  | 2021 | 39 | 99.24 ± 3.95 | 14.77 ± 1.44 | 14.97 ± 0.73 | 53 | 97.81 ± 3.66 | 14.15 ± 1.71 | 14.75 ± 1.10 |
|  | 2022 | 48 | 98.27 ± 3.52 | 15.00 ± 1.56 | 15.51 ± 1.08 | 31 | 97.53 ± 3.23 | 14.43 ± 1.49 | 15.13 ± 0.89 |
|  | *F* value |  | 1.834 | 0.953 | 1.861 |  | 3.119 | 3.152 | 3.674 |
|  | *P* value |  | 0.122 | 0.434 | 0.117 |  | 0.015 | 0.015 | 0.006 |
| 3.5- | Total | 1,323 | 101.97 ± 3.79 | 15.90 ± 1.83 | 15.26 ± 1.13 | 1,309 | 100.74 ± 3.79 | 15.27 ± 1.72 | 15.02 ± 1.13 |
|  | 2018 | 284 | 101.96 ± 3.62 | 15.83 ± 1.81 | 15.20 ± 1.19 | 302 | 100.70 ± 3.93 | 15.25 ± 1.72 | 15.01 ± 1.13 |
|  | 2019 | 173 | 102.69 ± 3.71 | 16.16 ± 1.67 | 15.29 ± 0.96 | 179 | 100.74 ± 3.91 | 15.34 ± 1.77 | 15.08 ± 1.10 |
|  | 2020 | 326 | 101.84 ± 3.91 | 16.00 ± 1.98 | 15.39 ± 1.23 | 322 | 100.54 ± 3.51 | 15.23 ± 1.63 | 15.05 ± 1.14 |
|  | 2021 | 286 | 101.84 ± 3.84 | 15.64 ± 1.68 | 15.05 ± 1.00 | 278 | 100.90 ± 4.00 | 15.24 ± 1.91 | 14.92 ± 1.18 |
|  | 2022 | 254 | 101.79 ± 3.82 | 15.94 ± 1.88 | 15.34 ± 1.17 | 228 | 100.85 ± 3.64 | 15.34 ± 1.55 | 15.06 ± 1.06 |
|  | *F* value |  | 1.885 | 2.661 | 3.984 |  | 0.400 | 0.229 | 0.759 |
|  | *P* value |  | 0.111 | 0.031 | 0.003 |  | 0.809 | 0.922 | 0.552 |
| 4.0- | Total | 2,048 | 104.82 ± 4.01 | 16.69 ± 2.01 | 15.15 ± 1.16 | 1,919 | 103.83 ± 4.01 | 16.12 ± 1.87 | 14.92 ± 1.12 |
|  | 2018 | 335 | 104.69 ± 3.99 | 16.59 ± 1.91 | 15.10 ± 1.09 | 338 | 103.84 ± 4.00 | 16.18 ± 1.93 | 14.98 ± 1.21 |
|  | 2019 | 366 | 104.87 ± 4.09 | 16.73 ± 1.97 | 15.18 ± 1.13 | 299 | 103.67 ± 4.07 | 16.05 ± 1.93 | 14.90 ± 1.13 |
|  | 2020 | 375 | 104.62 ± 4.08 | 16.64 ± 2.27 | 15.15 ± 1.30 | 333 | 103.60 ± 4.23 | 16.00 ± 1.76 | 14.88 ± 1.05 |
|  | 2021 | 519 | 105.12 ± 3.89 | 16.71 ± 1.97 | 15.09 ± 1.16 | 517 | 103.96 ± 3.84 | 15.99 ± 1.73 | 14.77 ± 1.04 |
|  | 2022 | 453 | 104.69 ± 4.04 | 16.72 ± 1.92 | 15.23 ± 1.12 | 432 | 103.97 ± 3.98 | 16.35 ± 1.99 | 15.09 ± 1.16 |
|  | *F* value |  | 1.193 | 0.363 | 1.082 |  | 0.669 | 2.831 | 5.151 |
|  | *P* value |  | 0.312 | 0.835 | 0.364 |  | 0.614 | 0.023 | <0.001 |
| 4.5- | Total | 2,210 | 108.39 ± 4.28 | 17.86 ± 2.31 | 15.15 ± 1.24 | 2,092 | 107.66 ± 4.19 | 17.38 ± 2.26 | 14.95 ± 1.28 |
|  | 2018 | 398 | 108.48 ± 4.17 | 17.84 ± 2.30 | 15.12 ± 1.23 | 341 | 107.57 ± 4.07 | 17.31 ± 2.16 | 14.92 ± 1.19 |
|  | 2019 | 434 | 108.33 ± 4.10 | 17.78 ± 2.11 | 15.12 ± 1.23 | 424 | 107.53 ± 4.01 | 17.35 ± 2.08 | 14.97 ± 1.28 |
|  | 2020 | 240 | 108.18 ± 4.53 | 17.89 ± 2.53 | 15.23 ± 1.42 | 246 | 107.82 ± 4.61 | 17.39 ± 2.76 | 14.88 ± 1.46 |
|  | 2021 | 667 | 108.65 ± 4.35 | 17.92 ± 2.41 | 15.12 ± 1.26 | 593 | 107.93 ± 4.09 | 17.32 ± 2.12 | 14.83 ± 1.16 |
|  | 2022 | 471 | 108.12 ± 4.28 | 17.84 ± 2.22 | 15.22 ± 1.15 | 488 | 107.40 ± 4.30 | 17.52 ± 2.37 | 15.14 ± 1.36 |
|  | *F* value |  | 1.291 | 0.260 | 0.819 |  | 1.307 | 0.713 | 4.467 |
|  | *P* value |  | 0.271 | 0.904 | 0.513 |  | 0.265 | 0.583 | 0.001 |
| 5.0- | Total | 2,073 | 111.90 ± 4.37 | 19.14 ± 2.62 | 15.24 ± 1.38 | 1,949 | 110.97 ± 4.40 | 18.46 ± 2.50 | 14.95 ± 1.34 |
|  | 2018 | 364 | 111.88 ± 4.41 | 19.15 ± 2.60 | 15.25 ± 1.32 | 342 | 111.02 ± 4.63 | 18.55 ± 2.75 | 14.99 ± 1.39 |
|  | 2019 | 329 | 111.91 ± 4.33 | 19.09 ± 2.59 | 15.19 ± 1.36 | 314 | 111.20 ± 4.30 | 18.62 ± 2.35 | 15.02 ± 1.33 |
|  | 2020 | 296 | 112.15 ± 4.44 | 19.31 ± 2.71 | 15.31 ± 1.48 | 285 | 111.17 ± 4.13 | 18.36 ± 2.39 | 14.81 ± 1.33 |
|  | 2021 | 518 | 111.88 ± 4.46 | 18.97 ± 2.60 | 15.11 ± 1.30 | 451 | 110.99 ± 4.65 | 18.30 ± 2.57 | 14.80 ± 1.33 |
|  | 2022 | 566 | 111.79 ± 4.25 | 19.22 ± 2.61 | 15.34 ± 1.44 | 557 | 110.68 ± 4.24 | 18.51 ± 2.40 | 15.07 ± 1.31 |
|  | *F* value |  | 0.329 | 1.020 | 2.187 |  | 0.978 | 1.070 | 3.607 |
|  | *P* value |  | 0.859 | 0.396 | 0.068 |  | 0.418 | 0.370 | 0.006 |
| 5.5- | Total | 2,339 | 115.46 ± 4.65 | 20.65 ± 3.13 | 15.43 ± 1.58 | 2,167 | 114.46 ± 4.59 | 19.77 ± 2.80 | 15.04 ± 1.44 |
|  | 2018 | 506 | 115.39 ± 4.48 | 20.50 ± 2.88 | 15.35 ± 1.49 | 460 | 114.41 ± 4.72 | 19.82 ± 2.83 | 15.09 ± 1.45 |
|  | 2019 | 339 | 115.27 ± 4.64 | 20.45 ± 3.13 | 15.32 ± 1.53 | 322 | 114.51 ± 4.44 | 19.66 ± 2.67 | 14.94 ± 1.32 |
|  | 2020 | 459 | 115.55 ± 4.60 | 20.69 ± 3.28 | 15.43 ± 1.72 | 457 | 114.28 ± 4.71 | 19.52 ± 2.83 | 14.90 ± 1.49 |
|  | 2021 | 345 | 116.19 ± 4.77 | 20.88 ± 3.01 | 15.41 ± 1.44 | 311 | 114.82 ± 4.83 | 19.79 ± 2.99 | 14.94 ± 1.45 |
|  | 2022 | 690 | 115.19 ± 4.71 | 20.73 ± 3.26 | 15.55 ± 1.63 | 617 | 114.43 ± 4.36 | 19.96 ± 2.70 | 15.20 ± 1.41 |
|  | *F* value |  | 2.931 | 1.247 | 1.846 |  | 0.676 | 1.812 | 3.884 |
|  | *P* value |  | 0.020 | 0.289 | 0.117 |  | 0.609 | 0.124 | 0.004 |
| 6.0- | Total | 2,120 | 118.76 ± 4.78 | 22.07 ± 3.55 | 15.58 ± 1.74 | 1,953 | 117.62 ± 4.87 | 21.03 ± 3.20 | 15.14 ± 1.58 |
|  | 2018 | 426 | 118.35 ± 4.84 | 21.94 ± 3.57 | 15.59 ± 1.75 | 419 | 117.20 ± 4.79 | 20.77 ± 3.17 | 15.06 ± 1.54 |
|  | 2019 | 318 | 118.98 ± 4.74 | 22.13 ± 3.40 | 15.57 ± 1.61 | 285 | 118.10 ± 4.73 | 21.09 ± 2.98 | 15.08 ± 1.54 |
|  | 2020 | 384 | 118.76 ± 5.22 | 22.28 ± 3.98 | 15.71 ± 1.99 | 387 | 117.38 ± 4.80 | 20.77 ± 3.15 | 15.02 ± 1.58 |
|  | 2021 | 447 | 119.35 ± 4.45 | 22.15 ± 3.36 | 15.49 ± 1.71 | 389 | 118.10 ± 4.91 | 21.12 ± 3.28 | 15.07 ± 1.57 |
|  | 2022 | 545 | 118.47 ± 4.67 | 21.93 ± 3.46 | 15.56 ± 1.66 | 473 | 117.50 ± 5.01 | 21.38 ± 3.29 | 15.42 ± 1.60 |
|  | *F* value |  | 3.176 | 0.767 | 0.874 |  | 2.738 | 2.833 | 4.916 |
|  | *P* value |  | 0.013 | 0.547 | 0.479 |  | 0.027 | 0.023 | <0.001 |
| 6.5- | Total | 1,118 | 121.46 ± 4.87 | 23.35 ± 4.01 | 15.75 ± 1.97 | 1,004 | 120.50 ± 4.85 | 22.11 ± 3.47 | 15.17 ± 1.69 |
|  | 2018 | 201 | 121.33 ± 5.07 | 23.09 ± 4.18 | 15.60 ± 2.07 | 163 | 120.40 ± 5.12 | 21.90 ± 3.26 | 15.04 ± 1.48 |
|  | 2019 | 234 | 121.42 ± 4.66 | 23.16 ± 3.42 | 15.65 ± 1.64 | 206 | 120.16 ± 4.84 | 21.90 ± 3.44 | 15.10 ± 1.59 |
|  | 2020 | 200 | 121.40 ± 4.90 | 23.97 ± 4.72 | 16.17 ± 2.43 | 174 | 120.53 ± 4.73 | 22.36 ± 4.14 | 15.31 ± 2.08 |
|  | 2021 | 313 | 121.59 ± 4.83 | 23.22 ± 3.94 | 15.63 ± 1.91 | 307 | 120.99 ± 4.68 | 22.16 ± 3.16 | 15.10 ± 1.66 |
|  | 2022 | 170 | 121.51 ± 5.01 | 23.46 ± 3.74 | 15.81 ± 1.69 | 154 | 120.06 ± 5.02 | 22.22 ± 3.53 | 15.34 ± 1.62 |
|  | *F* value |  | 0.108 | 1.664 | 3.106 |  | 1.375 | 0.612 | 1.117 |
|  | *P* value |  | 0.980 | 0.156 | 0.015 |  | 0.241 | 0.654 | 0.347 |

n: the number of annual health examination records; BMI: body mass index.

One-way ANOVA test among height, weight, and BMI from 2018 to 2022 in each age group; *P* < 0.05 indicates a statistically significant difference.

Table 3 Sex- and age-stratified comparison of overweight, obesity, and OwO prevalence across five years (N=17,561, n=26,246)

| Age | Year | **Boys (n=13,542)** | | | | **Girls (n=12,704)** | | | |
| --- | --- | --- | --- | --- | --- | --- | --- | --- | --- |
|  |  | number | Overweight, n (%) | Obesity, n (%) | OwO, n (%) | number | Overweight, n (%) | Obesity, n (%) | OwO, n (%) |
| 3.0- | Total | 311 | 19 (6.1) | 5 (1.6) | 24 (7.7) | 311 | 23 (7.4) | 1 (0.3) | 24 (7.7) |
|  | 2018 | 49 | 2 (4.1) | 0 (0.0) | 2 (4.1) | 50 | 7 (14.0) | 0 (0.0) | 7 (14.0) |
|  | 2019 | 55 | 3 (5.5) | 3 (5.5) | 6 (10.9) | 65 | 4 (6.2) | 0 (0.0) | 4 (6.2) |
|  | 2020 | 120 | 9 (7.5) | 2 (1.7) | 11 (9.2) | 112 | 4 (3.6) | 1 (0.9) | 5 (4.5) |
|  | 2021 | 39 | 1 (2.6) | 0 (0.0) | 1 (2.6) | 53 | 4 (7.5) | 0 (0.0) | 4 (7.5) |
|  | 2022 | 48 | 4 (8.3) | 0 (0.0) | 4 (8.3) | 31 | 4 (12.9) | 0 (0.0) | 4 (12.9) |
|  | *χ^2^* value |  | 1.780 | 4.415 | 3.290 |  | 7.191 | 3.194 | 5.860 |
|  | *P* value |  | 0.792 | 0.184 | 0.508 |  | 0.107 | 1.000 | 0.188 |
|  | Trend *χ^2^* value |  | 0.347 | 0.963 | 0.004 |  | 0.178 | 0.019 | 0.148 |
|  | *P* for trend |  | 0.571 | 0.373 | 1.000 |  | 0.715 | 0.892 | 0.721 |
| 3.5- | Total | 1,323 | 136 (10.3) | 13 (1.0) | 149 (14.6) | 1,309 | 125 (9.5) | 15 (1.1) | 140 (10.7) |
|  | 2018 | 284 | 25 (8.8) | 4 (1.4) | 29 (10.2) | 302 | 26 (8.6) | 3 (1.0) | 29 (9.6) |
|  | 2019 | 173 | 17 (9.8) | 0 (0.0) | 17 (9.8) | 179 | 18 (10.1) | 2 (1.1) | 20 (11.2) |
|  | 2020 | 326 | 40 (12.3) | 5 (1.5) | 45 (13.8) | 322 | 24 (7.5) | 5 (1.6) | 29 (9.0) |
|  | 2021 | 286 | 21 (7.3) | 0 (0.0) | 21 (7.3) * | 278 | 33 (11.9) | 2 (0.7) | 35 (12.6) |
|  | 2022 | 254 | 33 (13.0) | 4 (1.6) | 37 (14.6) | 228 | 24 (10.5) | 3 (1.3) | 27 (11.8) |
|  | *χ^2^* value |  | 6.811 | 7.320 | 9.950 |  | 3.986 | 1.167 | 2.741 |
|  | *P* value |  | 0.146 | 0.081 | 0.041 |  | 0.408 | 0.915 | 0.602 |
|  | Trend *χ^2^* value |  | 0.879 | 0.011 | 0.755 |  | 1.104 | 0.011 | 1.702 |
|  | *P* for trend |  | 0.348 | 0.922 | 0.385 |  | 0.293 | 0.927 | 0.301 |
| 4.0- | Total | 2,048 | 161 (7.9) | 34 (1.7) | 195 (9.5) | 1,919 | 164 (8.5) | 20 (1.0) | 184 (9.6) |
|  | 2018 | 335 | 22 (6.6) | 4 (1.2) | 26 (7.8) | 338 | 33 (9.8) | 5 (1.5) | 38 (11.2) |
|  | 2019 | 366 | 24 (6.6) | 8 (2.2) | 32 (8.7) | 299 | 24 (8.0) | 4 (1.3) | 28 (9.4) |
|  | 2020 | 375 | 36 (9.6) | 9 (2.4) | 45 (12.0) | 333 | 25 (7.5) | 3 (0.9) | 28 (8.4) |
|  | 2021 | 519 | 39 (7.5) | 6 (1.2) | 45 (8.7) | 517 | 35 (6.8) | 1 (0.2) | 36 (7.0) |
|  | 2022 | 453 | 40 (8.8) | 7 (1.5) | 47 (10.4) | 432 | 47 (10.9) | 7 (1.6) | 54 (12.5) |
|  | *χ^2^* value |  | 3.872 | 3.167 | 4.956 |  | 6.300 | 7.013 | 9.953 |
|  | *P* value |  | 0.424 | 0.530 | 0.292 |  | 0.178 | 0.115 | 0.041 |
|  | Trend *χ^2^* value |  | 1.452 | 0.093 | 0.944 |  | 0.062 | 0.266 | 0.003 |
|  | *P* for trend |  | 0.228 | 0.760 | 0.331 |  | 0.803 | 0.635 | 0.953 |
| 4.5- | Total | 2,210 | 198 (9.0) | 13 (1.9) | 241 (10.9) | 2,092 | 192 (9.2) | 56 (2.7) | 248 (11.9) |
|  | 2018 | 398 | 35 (8.8) | 6 (1.5) | 41 (10.3) | 341 | 30 (8.8) | 5 (1.5) | 35 (10.3) |
|  | 2019 | 434 | 46 (10.6) | 5 (1.2) | 51 (11.8) | 424 | 35 (8.3) | 11 (2.6) | 46 (10.8) |
|  | 2020 | 240 | 18 (7.5) | 7 (2.9) | 25 (10.4) | 246 | 19 (7.7) | 12 (4.9) | 31 (12.6) |
|  | 2021 | 667 | 63 (9.4) | 17 (2.5) | 80 (12.0) | 593 | 50 (8.4) | 8 (1.3) * | 58 (9.8) |
|  | 2022 | 471 | 36 (7.6) | 8 (1.7) | 44 (9.3) | 488 | 58 (11.9) | 20 (4.1) | 78 (16.0) |
|  | *χ^2^* value |  | 3.264 | 4.441 | 2.527 |  | 5.804 | 14.303 | 11.77 |
|  | *P* value |  | 0.515 | 0.350 | 0.640 |  | 0.214 | 0.006 | 0.019 |
|  | Trend *χ^2^* value |  | 0.607 | 0.864 | 0.091 |  | 2.303 | 1.935 | 4.203 |
|  | *P* for trend |  | 0.436 | 0.353 | 0.762 |  | 0.129 | 0.164 | 0.040 |
| 5.0- | Total | 2,073 | 201 (9.7) | 54 (2.6) | 255 (12.3) | 1,949 | 188 (9.6) | 41 (2.1) | 229 (11.7) |
|  | 2018 | 364 | 37 (10.2) | 8 (2.2) | 45 (12.4) | 342 | 26 (7.6) | 11 (3.2) | 37 (10.8) |
|  | 2019 | 329 | 33 (10.0) | 8 (2.4) | 41 (12.5) | 314 | 37 (11.8) | 6 (1.9) | 43 (13.7) |
|  | 2020 | 296 | 32 (10.8) | 9 (3.0) | 41 (13.9) | 285 | 30 (10.5) | 3 (1.1) | 33 (11.6) |
|  | 2021 | 518 | 40 (7.7) | 9 (1.7) | 49 (9.5) | 451 | 37 (8.2) | 7 (1.6) | 44 (9.8) |
|  | 2022 | 566 | 59 (10.4) | 20 (3.5) | 79 (14.0) | 557 | 58 (10.4) | 14 (2.5) | 72 (12.9) |
|  | *χ^2^* value |  | 3.201 | 3.958 | 5.986 |  | 4.990 | 4.762 | 3.911 |
|  | *P* value |  | 0.525 | 0.412 | 0.200 |  | 0.288 | 0.313 | 0.418 |
|  | Trend *χ^2^* value |  | 0.110 | 0.818 | 0.020 |  | 0.237 | 0.281 | 0.044 |
|  | *P* for trend |  | 0.741 | 0.366 | 0.888 |  | 0.627 | 0.596 | 0.834 |
| 5.5- | Total | 2,339 | 258 (11.0) | 90 (3.8) | 348 (14.9) | 2,167 | 256 (11.8) | 53 (2.4) | 309 (14.3) |
|  | 2018 | 506 | 59 (11.7) | 12 (2.4) * | 71 (14.0) | 460 | 58 (12.6) | 12 (2.6) | 70 (15.2) |
|  | 2019 | 339 | 33 (9.7) | 8 (2.4) * | 41 (12.1) | 322 | 36 (11.2) | 4 (1.2) | 40 (12.4) |
|  | 2020 | 459 | 39 (8.5) | 30 (6.5) | 69 (15.0) | 457 | 47 (10.3) | 12 (2.6) | 59 (12.9) |
|  | 2021 | 345 | 42 (12.2) | 9 (2.6) * | 51 (14.8) | 311 | 40 (12.9) | 6 (1.9) | 46 (14.8) |
|  | 2022 | 690 | 85 (12.3) | 31 (4.5) | 116 (16.8) | 617 | 75 (12.2) | 19 (3.1) | 94 (15.2) |
|  | *χ^2^* value |  | 5.414 | 16.182 | 4.408 |  | 1.826 | 3.454 | 2.467 |
|  | *P* value |  | 0.247 | 0.003 | 0.354 |  | 0.768 | 0.485 | 0.651 |
|  | Trend *χ^2^* value |  | 0.734 | 3.025 | 2.871 |  | 0.010 | 0.631 | 0.194 |
|  | *P* for trend |  | 0.391 | 0.082 | 0.090 |  | 0.922 | 0.427 | 0.659 |
| 6.0- | Total | 2,120 | 267 (12.6) | 94 (4.4) | 361 (17.0) | 1,953 | 238 (12.2) | 62 (3.2） | 300 (15.4) |
|  | 2018 | 426 | 52 (12.2) | 21 (4.9) | 73 (17.1) | 419 | 50 (11.9) | 12 (2.9) | 62 (14.8) |
|  | 2019 | 318 | 41 (12.9) | 10 (3.1) * | 51 (16.0) | 285 | 26 (9.1) | 9 (3.2) | 35 (12.3) |
|  | 2020 | 384 | 42 (10.9) | 30 (7.8) | 72 (18.8) | 387 | 43 (11.1) | 11 (2.8) | 54 (14.0) |
|  | 2021 | 447 | 62 (13.9) | 15 (3.4) * | 77 (17.2) | 389 | 49 (12.6) | 11 (2.8) | 60 (15.4) |
|  | 2022 | 545 | 70 (12.8) | 18 (3.3) * | 88 (16.1) | 473 | 70 (14.8) | 19 (4.0) | 89 (18.8) |
|  | *χ^2^* value |  | 1.733 | 14.711 | 1.342 |  | 6.021 | 1.515 | 7.116 |
|  | *P* value |  | 0.785 | 0.005 | 0.854 |  | 0.198 | 0.824 | 0.130 |
|  | Trend *χ^2^* value |  | 0.230 | 1.535 | 0.065 |  | 3.058 | 0.662 | 3.929 |
|  | *P* for trend |  | 0.631 | 0.215 | 0.799 |  | 0.080 | 0.416 | 0.047 |
| 6.5- | Total | 1,118 | 155 (13.9) | 57 (5.1) | 212 (19.0) | 1,004 | 138 (13.7) | 33 (3.3) | 171 (17.0) |
|  | 2018 | 201 | 24 (11.9) | 11 (5.5) | 35 (17.4) | 163 | 21 (12.9) | 3 (1.8) | 24 (14.7) |
|  | 2019 | 234 | 43 (18.4) | 3 (1.3) * | 46 (19.7) | 206 | 31 (15.0) | 4 (1.9) | 35 (17.0) |
|  | 2020 | 200 | 29 (14.5) | 21 (10.5) | 50 (25.0) | 174 | 22 (12.6) | 11 (6.3) | 33 (19.0) |
|  | 2021 | 313 | 42 (13.4) | 15 (4.8) | 57 (18.2) | 307 | 38 (12.4) | 10 (3.3) | 48 (15.6) |
|  | 2022 | 170 | 17 (10.0) | 7 (4.1) | 24 (14.1) | 154 | 26 (16.9) | 5 (3.2) | 31 (20.1) |
|  | *χ^2^* value |  | 6.857 | 19.561 | 7.844 |  | 2.338 | 7.289 | 2.545 |
|  | *P* value |  | 0.144 | 0.001 | 0.097 |  | 0.674 | 0.121 | 0.637 |
|  | Trend *χ^2^* value |  | 1.241 | 0.100 | 0.648 |  | 0.149 | 0.937 | 0.659 |
|  | *P* for trend |  | 0.265 | 0.752 | 0.421 |  | 0.700 | 0.333 | 0.417 |

OwO: combined overweight and obesity; N: number of children; n: the number of annual health examination records.

*P* values are unadjusted and were derived from Pearson’s chi-square test for overall between-year comparisons within each sex and age group.
*P* for trend was calculated using the Mantel-Haenszel test for trend.

* *P* < 0.0125, compared with 2020 after Bonferroni correction for pre-specified pairwise comparisons.
